# Supplementary material for: Multidisciplinary aerodigestive program at a children’s hospital: A protocol for a prospective observational study
Source: PLoS One. 2021 Oct 27;16(10):e0259208. doi: 10.1371/journal.pone.0259208 (PMC8550601; doi:10.1371/journal.pone.0259208)
Supplement: S1 Table — (DOCX) [file pone.0259208.s001.docx]

# S1 Table. The form of interview for subjective symptom scoring

| **Sex / Age** |  |
| --- | --- |
| **Primary diagnosis** |  |
| **Reason for consult** |  |
| **Feeding route** | □ Oral feeding □ Enteral tube □ Gastrostomy □ Others ( ) |
| **Respiratory support** | □ None □ Oxygen supply (__________hr(s)/day or __________hr(s)/week)  □ Home ventilation via nasal or facial mask (__________hr(s)/day or __________hr(s)/week)  □ Home ventilation via tracheostomy (__________hr(s)/day or __________hr(s)/week) |

| **Main Symptom**^a^ | **Importance**  (1-10^b^) | **MM-DD-YY (Before)** | | **MM-DD-YY (After)** | |
| --- | --- | --- | --- | --- | --- |
|  |  | **Severity**  (1-10^b^) | **Satisfaction**  (1-10^b^) | **Severity**  (1-10^b^) | **Satisfaction**  (1-10^b^) |
| 1. |  |  |  |  |  |
| 2. |  |  |  |  |  |
| 3. |  |  |  |  |  |
| 4. |  |  |  |  |  |
| 5. |  |  |  |  |  |
| **Average** |  |  |  |  |  |

a. Use one of the expression on Table 2.

b. 1 to 10 points, 1 point is the least and 10 point is the most
