# Supplementary material for: Multidisciplinary aerodigestive program at a children’s hospital: A protocol for a prospective observational study
Source: PLoS One. 2021 Oct 27;16(10):e0259208. doi: 10.1371/journal.pone.0259208 (PMC8550601; doi:10.1371/journal.pone.0259208)
Supplement: Attachment — Submitted filename: ADT_Response to reviewers_20210823.docx [file pone.0259208.s002.docx]

**Point-by-Point Responses to the Comments**

We thank the editor and reviewers for their constructive comments and review of the content. We have attempted to address all the concerns raised by the reviewers and have incorporated the relevant changes in the manuscript, where they are highlighted in yellow. Point-by-point responses to the reviewers’ comments are given below.

**Reviewer 1**

**Aerodigestive programs to enhance and coordinate the management of complex children are desirable and increasingly common. However good evaluation of the value of these programs to patients is still lacking. This is the first prospective evaluation of a multidisciplinary program that I am aware of, and I applaud the effort. My observation, however, is that in most programs, pulmonary and gastroenterology are key components. It appears to me that there are over 20 authors, and yet none is a pulmonologist or gastroenterologist. This may be a consideration?**

🡪 Thank you for the positive feedback and valuable comments. We agree that pediatric pulmonology and gastroenterology are important components and have mentioned that the pediatric pulmonologist and pediatric gastroenterologist should be core members of the aerodigestive program in the “Study design and setting” section. Moreover, the authors include four pediatric pulmonologists, namely Soo Yeon Kim, Min Jung Kim, Myung Hyun Sohn, and myself (a corresponding author), and three pediatric gastroenterologists, namely Seung Kim, Sowon Park, and Hong Koh. We apologize for not indicating the specific divisions in the affiliations. We have now rectified this issue, as shown below.

**Authors’ affiliations**

Mireu Park, M.D.*^1^, Seung Kim, M.D., M.S.^2^, Eunyoung Kim, M.S.N.^3^, Ga Eun Kim, M.D.^1^, Jae Hwa Jung, M.D.^1^, Soo Yeon Kim, M.D.^1^, Min Jung Kim, M.D., Ph.D.^1^, Da Hee Kim, M.D.^4^, Sowon Park, M.D.^2^, In Geol Ho, M.D.^5^, Seung Ki Kim, M.D.^6^, Sangwon Hwang, M.D.^7^, Kyeong Hun Shin, R.D.^8^, Hosun Lee, R.D.^8^, Bobae Lee, B.S.N.^2^, Hyeyeon Lee, OTR. B.H.S.^9^, Minhwa Park, OTR. B.H.S.^9^, Hong Koh, M.D., Ph.D.^2^, Myung Hyun Sohn, M.D., Ph.D.^1^, Dong-Wook Rha, M.D., Ph.D.^7^, Kyung Won Kim, M.D., Ph.D.^1^

^1^Division of Allergy, Respiratory and Critical Care Medicine, Department of Pediatrics, Severance Hospital, Yonsei University College of Medicine, Seoul, Korea

^2^Division of Gastroenterology, Hepatology and Nutrition, Department of Pediatrics, Severance Hospital, Yonsei University College of Medicine, Seoul, Korea

^3^Department of Nursing, Severance Children Hospital, Yonsei University Health System

^4^Department of Otorhinolaryngology, Yonsei University College of Medicine, Seoul, Korea

^5^Department of Pediatric Surgery, Severance Children’s Hospital, Yonsei University College of Medicine, Seoul, Korea

^6^Department of Rehabilitation Medicine, Yongin Severance Hospital, Research Institute of Rehabilitation Medicine, Yonsei University College of Medicine, Seoul, Korea.

^7^Department and Research Institute of Rehabilitation Medicine, Yonsei University College of Medicine, Seoul, Korea

^8^Department of Nutrition Care, Severance Hospital, Yonsei University Health System

^9^Department of Pediatric Occupational Therapy, Severance Rehabilitation hospital, Yonsei University Health System

**Reveiwer 2**

**Is the 1-10 score an visual analogue score or a subjective score by parents of a number scale? Besides this point, the protocol of a prospective observational study is entirely appropriate.**

🡪 Thank you for the positive feedback, and we apologize for the lack of clarity regarding the score. The scores from 1 to 10 were subjectively assigned by the parents using a number scale. Most patients referred to the aerodigestive program cannot communicate about their uncomfortable symptoms due to chronic complex medical conditions. Sometimes, subtle changes and expressions are often difficult to interpret by anyone other than the primary caregivers. Moreover, we considered that subjective scoring by the primary caregiver, rather than recognition of the condition at one point in time by medical staff using the visual analog score, would be a more accurate subjective outcome measure. Furthermore, more accurate evaluation would be possible if the subjective evaluation category was divided into severity and satisfaction rather than a single category. To eliminate the ambiguity, we have made changes in the manuscript as shown below.

**Study process**

The importance of each major symptom was scored from 1 to 10 points and prioritized. Also, the patient evaluated the severity and satisfaction of each symptom by comparing the scores before and after the treatment.

🡪 The importance of each major symptom was scored from 1 to 10 points and prioritized by the patients and their primary caregivers, who also evaluated the severity and satisfaction of each symptom by comparing the scores before and after the treatment.

**Reviewer 3**

**1. Does the ADT meeting includes psychologists, social assistants or specialty nurses in addition to physicians?**

🡪 Thank you for your pertinent question. Psychologists and social assistants are not included at this point, although we agree that the patients and their families can receive a wider range of care, including psychological and financial support, if they are included in the team. Therefore, we have plans to include psychologists and social assistants as members of our program soon.

🡪 An advanced nurse specialized in pediatrics is included as a core member and care coordinator who participates in all stages of the ADT program. The specialty nurse in our ADT program is Eunyoung Kim, mentioned in the authors’ list.

**2. If interventions are planned, do you intent to do it in a single operative time or each specialty plans their own interventions? For example, if a child has indication to underwent a microlaryngoscopy, a bronchoscopy and an upper digestive endoscopy - are these done at the same surgical time or in separate times? Please justify your answer**.

🡪 Thank you for the valuable comments. Our hospital has separate rooms for procedures such as laryngoscopy, bronchoscopy, and esophagastroduodenoscopy under light sedation. Laryngomicrosurgery and gastrostomy with fundoplication are performed in the operating room under general anesthesia. However, these surgical procedures are personalized and usually performed in a step-by-step fashion, not at the same surgical time. However, if it is necessary to perform these procedures at the same time, we do the needful in order to avoid multiple separate procedures under sedation or anesthesia.

**3. In which situations would it be beneficial to perform a neck x-ray? Please justify your answer.**

🡪 Neck X-rays are obtained in the anteroposterior and lateral positions for screening of obstruction or narrowing of the upper respiratory tract and for evaluation of tracheal deviation. Moreover, for patients who are under consideration for tracheostomy, neck X-rays are beneficial to check the length and gross anatomy of the neck. In addition, after surgical intervention, we acquire neck X-rays to check whether the upper airway structure or the tracheostomy position is well maintained.

**4. References number 2 and 7 are incomplete**

🡪 We apologize for this oversight. We have rectified the references as shown below.

**In the References**

2.	Acra S, Cofer SA, Boesch RP, Dahl JP, Daines CL, Dealarcon A, et al. Structure and Functions of Pediatric Aerodigestive Programs: A Consensus Statement.

🡪 2.	Boesch RP, Balakrishnan K, Acra S, Benscoter DT, Cofer SA, Collaco JM, et al. Structure and Functions of Pediatric Aerodigestive Programs: A Consensus Statement. Pediatrics. 2018;141(3).

7.	Anand I, Pentiuk S, Garza J, Su W, Fei L, Kaul A. GER characteristics after fundoplication in children with aerodigestive disorders. Journal of pediatric surgery. 2019.

🡪 7.	Anand I, Pentiuk S, Garza J, Su W, Fei L, Kaul A. GER characteristics after fundoplication in children with aerodigestive disorders. J Pediatr Surg. 2020;55(3):456-60.

**5. Line 173: please correct the word "laryngomicrosurgery"**

🡪 Thank you for pointing this out. We have corrected the term in the revised manuscript.

**Study process**

The surgical intervention including layngomicrosurgery, primary dilatation for the airway, tracheostomy, gastrostomy, fundoplication surgery, percutaneous endoscopic gastrostomy, etc., and the name, date, and other specific operative findings were recorded.

🡪 The surgical intervention, which included laryngomicrosurgery, primary dilatation for the airway, tracheostomy, gastrostomy, fundoplication surgery, and percutaneous endoscopic gastrostomy, among others, as well as the name, date, and other specific operative findings, were recorded.
